# Oscillometry and pulmonary magnetic resonance imaging in asthma and COPD

**DOI:** 10.14814/phy2.13955

**Published:** 2019-01-10

**Authors:** Rachel L. Eddy, Andrew Westcott, Geoffrey N. Maksym, Grace Parraga, Ronald J. Dandurand

**Affiliations:** ^1^ Robarts Research Institute London Ontario Canada; ^2^ Department of Medical Biophysics Western University London Ontario Canada; ^3^ School of Biomedical Engineering Dalhousie University Halifax Nova Scotia Canada; ^4^ CIUSSS de l'Ouest‐de‐l’Île‐de‐Montréal, Montreal Chest Institute, Meakins‐Christie Laboratories, Oscillometry Unit and Centre for Innovative Medicine McGill University Health Centre and Research Institute Montreal Quebec Canada

**Keywords:** Asthma, COPD, MRI, oscillometry

## Abstract

Developed over six decades ago, pulmonary oscillometry has re‐emerged as a noninvasive and effort‐independent method for evaluating respiratory‐system impedance in patients with obstructive lung disease. Here, we evaluated the relationships between hyperpolarized ^3^He ventilation‐defect‐percent (VDP) and respiratory‐system resistance, reactance and reactance area (A_X_) measurements in 175 participants including 42 never‐smokers without respiratory disease, 56 ex‐smokers with chronic‐obstructive‐pulmonary‐disease (COPD), 28 ex‐smokers without COPD and 49 asthmatic never‐smokers. COPD participants were dichotomized based on x‐ray computed‐tomography (CT) evidence of emphysema (relative‐area CT‐density‐histogram ≤ 950HU (RA_950_) ≥ 6.8%). In asthma and COPD subgroups, MRI VDP was significantly related to the frequency‐dependence of resistance (*R*_5‐19_; asthma: *ρ *= 0.48, *P* = 0.0005; COPD:* ρ *= 0.45, *P* = 0.0004), reactance at 5 Hz (*X*_5_: asthma, *ρ *= −0.41, *P* = 0.004; COPD:* ρ *= −0.38, *P* = 0.004) and *A*_*X*_ (asthma: *ρ *= 0.47, *P* = 0.0007; COPD:* ρ *= 0.43, *P* = 0.0009). MRI VDP was also significantly related to *R*_5‐19_ in COPD participants without emphysema (*ρ *= 0.54, *P* = 0.008), and to *X*_5_ in COPD participants with emphysema (*ρ *= −0.36, *P* = 0.04). *A*_*X*_ was weakly related to VDP in asthma (*ρ *= 0.47, *P* = 0.0007) and COPD participants with (*ρ *= 0.39, *P* = 0.02) and without (*ρ *= 0.43, *P* = 0.04) emphysema. *A*_*X*_ is sensitive to obstruction but not specific to the type of obstruction, whereas the different relationships for MRI VDP with *R*_5‐19_ and *X*_5_ may reflect the different airway and parenchymal disease‐specific biomechanical abnormalities that lead to ventilation defects.

## Introduction

First developed over 60 years ago (Dubois et al. 1956), oscillometry has re‐emerged as a way to generate clinical measurements in patients with obstructive lung disease because minimal coaching and patient effort is required. Moreover, oscillometry is well‐tolerated in young and old patients across disease severities (Smith et al. 2005) and is sensitive to small airway abnormalities (Peslin and Fredberg 1986). Oscillometry also provides a noninvasive way (Oostveen et al. 2003) to reveal lung pathologies that result in ventilation heterogeneity (Otis et al. 1956; Lutchen and Gillis 1997; Kaczka et al. 2011) by directly measuring resistance and reactance as functions of frequency. It is well established that in asthma, respiratory‐system resistance responds to bronchodilator inhalation (van Noord et al. 1994; Zerah et al. 1995; Kaczka et al. 1999; Delacourt et al. 2000) and is frequency‐dependent (Grimby et al. 1968; Bhansali et al. 1979; Brochard et al. 1987). The frequency dependence of resistance has also been observed in patients with chronic obstructive pulmonary disease (COPD) (di Mango et al. 2006), in whom low‐frequency resistance also diminishes after bronchodilation (van Noord et al. 1994; Zerah et al. 1995).

In patients with asthma and COPD, reactance is more negative at low frequencies (Clement et al. 1983). The area under the reactance curve can be quantified as the reactance area (*A*_*X*_) (Goldman 2001) which is determined by the reactance value measured at the lowest frequency, the resonant frequency, and the shape of the low‐frequency reactance curve. *A*_*X*_ measurements correlate strongly with the frequency dependence of resistance (Skloot et al. 2004) and in asthmatics, *A*_*X*_ detects bronchodilator (van Noord et al. 1994) and bronchial challenge (van Noord et al. 1989) responses in the absence of low‐frequency reactance changes. Furthermore, A_X_ has been suggested as a useful tool for early disease screening and monitoring in COPD, and may be more sensitive to therapy response than the frequency dependence of resistance (Lipworth and Jabbal 2018).

X‐ray computed tomography (CT) airway measurements were previously shown to be related to oscillometry measurements of resistance in asthma (Karayama et al. 2018) and COPD (Karayama et al. 2017). Quantitative CT measurements of emphysema have also been shown to be related to oscillometry‐measured reactance in COPD (Karayama et al. 2017) and there are differences in the relationships between CT measurements and respiratory impedance in different COPD phenotypes (Wada et al. 2018). Magnetic resonance imaging (MRI) using inhaled noble gases was also recently used to discern the relationships between low‐frequency resistance and elastance as well as the frequency dependence of resistance with MRI signal intensity coefficients of variation (Lui et al. 2015). Another study showed a relationship between MRI ventilation defect percent (VDP) and the frequency‐dependence of resistance in COPD patients (Young et al. 2018).

While these previous results are intriguing, no large‐scale, controlled study has investigated a diversity of patients across a spectrum of disease severities to ascertain the relationships between experimental oscillometry measurements and imaging biomarkers of airway and parenchymal disease. This is important because in patients with asthma and COPD, airway and parenchymal abnormalities both contribute to symptomatic derangements in lung function and poor exercise capacity. In recent years, there has been modest clinical support for experimental impedance measurements as a way to evaluate patients (Shi et al. 2012; Lipworth and Jabbal 2018). Accordingly, our objective was to investigate the relationships between oscillometry measurements including resistance, reactance and the frequency dependence of resistance as well as A_X_ with MRI ventilation defect measurements across a wide variety of patients. In contrast with previous investigations (Lui et al. 2015; Young et al. 2018), here we evaluated participants with asthma and those with COPD (with and without emphysema) as well as control groups of never‐smokers without asthma and ex‐smokers without COPD.

## Materials and Methods

### Study participants and design

We evaluated never‐smokers aged 60–90 years, asthmatics aged 18–70 years and ex‐smokers with and without COPD aged 50–90 years who provided written informed consent to study protocols approved by the local research ethics board and Health Canada and registered (NCT02483403, NCT02279329, NCT02351141 https://clinicaltrials.gov). All subjects underwent a single three‐hour study visit including spirometry, plethysmography, oscillometry, and MRI. Some of these subjects were previously evaluated and results published (Young et al. 2018). Never‐smokers performed all testing without administration of a short‐acting bronchodilator. Participants with asthma and all ex‐smokers performed all testing after administration of a short‐acting bronchodilator. In addition, all ex‐smokers underwent post‐bronchodilator thoracic CT. Post‐bronchodilator testing was performed 20 min after administration of four inhaled doses of 100 *μ*g Novo‐Salbutamol HFA (Teva Novopharm Ltd., Toronto, ON, Canada) through a pressurized metered‐dose inhaler using an *AeroChamber Plus* spacer (Trudell Medical International, London, ON, Canada).

### Pulmonary function tests

Spirometry and plethysmography were performed using a *MedGraphics Elite Series* plethysmograph (MGC Diagnostics Corporation, St. Paul, MN, USA). Spirometry was performed according to American Thoracic Society (ATS)/European Respiratory Society (ERS) guidelines (Miller et al. 2005) to measure the forced expiratory volume in one‐second (FEV_1_), forced vital capacity (FVC) and FEV_1_/FVC, while plethysmography was performed to measure lung volumes and airways resistance (*R*_aw_). For never‐smokers and all ex‐smokers, the diffusing capacity of the lung for carbon monoxide (DL_CO_) was also measured using a stand‐alone gas analyzer attached to the plethysmograph. For post‐bronchodilator testing in asthma, ex‐smoker and COPD subgroups, participants withheld short‐acting *β*‐agonists for 6 h, long‐acting *β*‐agonists for 12 h and long‐acting muscarinic antagonists for 24 h before their study visit.

### Oscillometry

Oscillometry was performed using the *tremoFlo C‐100* Airwave Oscillometry System (Thorasys, Montreal, QC, Canada) with the non‐harmonic composite airwaves in the adult frequency range consisting of 5, 11, 13, 17, 19, 23, 29, 21, and 37 Hz to measure total respiratory system resistance at 5 Hz (*R*_5_), frequency‐dependence of resistance as *R* at 5 Hz minus *R* at 19 Hz (*R*_5‐19_), reactance at 5 Hz (*X*_5_), resonant frequency (*f*_res_) and *A*_*X*_. *A*_*X*_ was calculated by integrating the reactance curve from 5 Hz to *f*_res_ and when *f*_res_ was greater than 37 Hz, the reactance curve was truncated at 37 Hz and integrated up to that point. Participants were seated comfortably with legs uncrossed and supported their chin and cheeks with their hands to limit upper airway shunt. Oscillometry measurements were acquired over 16 sec and repeated for three acceptable and repeatable tests, as judged by a coefficient of variation in resistance at 5 Hz (*CV*_R5_) of <15%. Artefacts were automatically identified and removed by the manufacturer's automated algorithms. Calibration of the oscillometry unit was performed daily using the vendor‐provided nominal 2 cmH_2_O·s/L reference test load.

### Image acquisition and analysis

All subjects underwent anatomical proton (^1^H) followed by hyperpolarized ^3^He static ventilation MRI (within 5 min) using a whole body 3T system (MR750 Discovery, General Electric Healthcare, Milwaukee, WI) with broadband imaging capability as previously described (Parraga et al. 2007). ^3^He gas was polarized to 30–40% polarization (HeliSpin; Polarean Inc., Durham, NC, USA) and diluted with N_2_ gas to 25% ^3^He by volume. Subjects were positioned supine in the scanner with their arms above their head and instructed to inhale 1.0 L of gas (100% N_2_ for ^1^H MRI, ^3^He/N_2_ mixture for ^3^He MRI) from functional residual capacity (FRC) and coronal images were acquired in 8–15 sec under breath‐hold conditions. For all image acquisition, FRC was assumed to be the lung volume at end tidal expiration.

Hyperpolarized ^3^He MR images were analyzed using in‐house segmentation software as previously described (Guo et al. 2015). Briefly, a single user placed seeds on the ^1^H and ^3^He images to label the lung and the surrounding background tissue and image registration and segmentation were completed automatically. ^3^He images were segmented into five clusters of signal intensity using three‐dimensional *k*‐means clustering (Kirby et al. 2012), and the ventilation defect percent (VDP) was quantified as the ventilation defect volume normalized to the thoracic cavity volume.

Ex‐smoker participants were transported from the MRI suite to the CT suite by wheelchair to avoid exercise‐induced dilatation of the airways. Thoracic CT volumes were acquired within 10 min of completion of MRI using a 64‐slice LightSpeed VCT system (General Electric Healthcare) as previously described (Owrangi et al. 2013) under breath‐hold conditions after full inspiration. The total effective dose for each CT scan was 1.8 mSv as calculated using the manufacturer's settings and the ImPACT patient dosimetry calculator (based on the UK Health Protection Agency NRPB‐SR250 software).

Thoracic CT images were analyzed using Pulmonary Workstation 2.0 (VIDA Diagnostics Inc., Coralville, IA, USA) to quantify emphysema using the relative area of the lung <−950 Hounsfield units (RA_950_). An RA_950_ threshold of 6.8% was used to stratify COPD subjects with and without CT evidence of emphysema such that RA_950_ ≥ 6.8% defined presence of emphysema (Gevenois et al. 1996).

### Statistical analysis

Data were tested for normality using the Shapiro‐Wilk test using IBM SPSS Statistics 25.0 (IBM Corporation, Armonk, NY, USA) and when not normally distributed, nonparametric statistics were performed. One‐way ANOVA and Kruskal–Wallis H test were performed for group‐wise differences with post‐hoc least significant difference and Holm‐Bonferroni correction to adjust for multiple comparisons and Fisher's exact test was used for categorical variables using SPSS. Univariate relationships were evaluated using Pearson correlations (*r*) for normally distributed data and Spearman correlations (*ρ*) when the data were not normally distributed using GraphPad Prism 7.00 (GraphPad Software, La Jolla, CA, USA). Multivariable models were generated in SPSS using the enter approach to determine the contributions of *R*_5_, *R*_5‐19_, *X*_5_, and *A*_*X*_ to VDP using age, sex and body mass index (BMI) as covariates for four separate models: (1) all subjects, (2) never‐smokers and ex‐smokers with and without COPD, (3) ex‐smokers with and without COPD, and (4) asthmatics only. Results were considered statistically significant when the probability of making a Type I error was less than 5% (*P* < 0.05).

## Results

**Figure 1:**
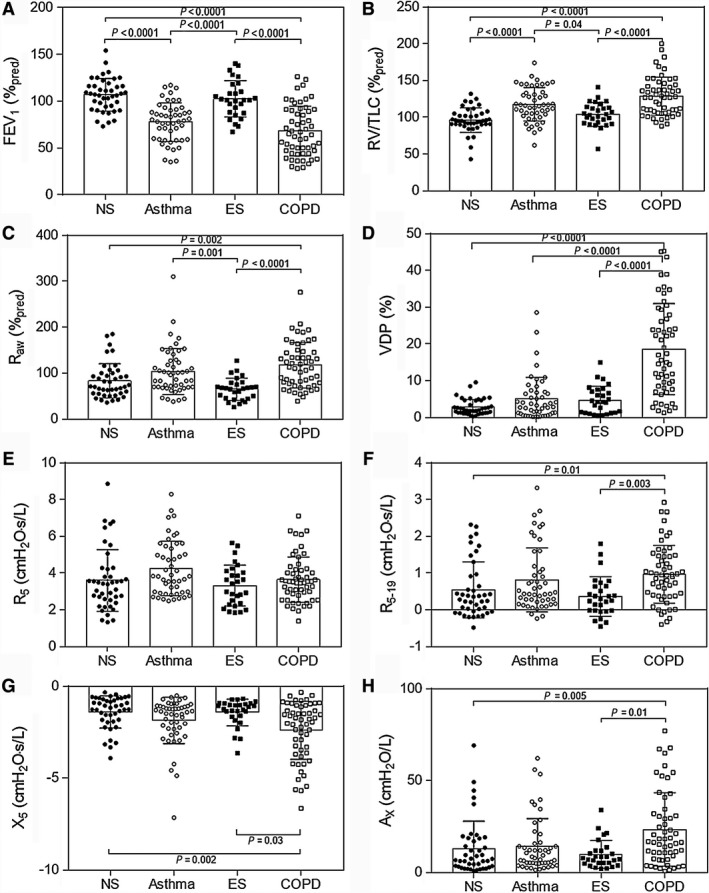
Pulmonary function test and MRI VDP measurements. (A) Significantly lower FEV_1_ in asthma and COPD compared to never‐smokers and ex‐smokers. (B) Significantly greater RV/TLC in asthma and COPD compared to never‐smokers and ex‐smokers. (C) Significantly greater *R*_aw_ in asthma as compared to ex‐smokers and COPD subjects and significantly greater *R*_aw_ in COPD as compared to never and ex‐smokers. (D) Significantly greater VDP in COPD as compared to all other subgroups. (E) *R*_5_ not significantly different between all subgroups. (F) Significantly greater *R*_5‐19_, and, (G) Significantly more negative *X*_5_, and, (H) significantly greater *A*_*X*_ in COPD as compared to never‐ and ex‐smokers.

We evaluated 175 participants including 42 elderly never‐smokers (74 ± 7 years), 49 participants with asthma (48 ± 12 years; *n* = 14 treatment steps 1–2, *n *= 35 treatment steps 3–4 as per the Global Initiative for Asthma [GINA] guidelines (Global Initiative for Asthma (GINA), 2017)), 28 ex‐smokers without COPD (70 ± 9 years) and 56 ex‐smokers with COPD (73 ± 9 years; *n* = 18 mild [GOLD I], *n* = 22 moderate [GOLD II], *n* = 16 severe [GOLD III‐IV]). Table 1 shows demographic, pulmonary function test and imaging measurements for never‐smokers, asthma participants, ex‐smokers, and COPD participants and between‐group differences are shown in Figure 1 for select data. We note that for 10 participants, *f*_res_ was greater than 37 Hz (*n* = 1 asthma, *n* = 9 COPD). Participants with COPD had significantly worse post‐bronchodilator pulmonary function than never‐smoker and ex‐smoker participants, whereas participants with asthma did not have significantly different post‐bronchodilator oscillometry measurements than never‐smokers and ex‐smokers. There were no significant differences between never‐smoker and ex‐smoker subgroups.

**Table 1:** Subject demographics

Parameter mean (±SD)	Never‐smokers (*n* = 42)	Asthma (*n* = 49)	Ex‐smokers (*n* = 28)	COPD (*n* = 56)	Sig diff* (*P*)
Age, years	74 (7)	48 (13)	70 (9)	73 (9)	**<0.0001**
Male, *n* (%)	21 (50)	19 (39)	16 (57)	36 (64)	0.1
BMI, kg/m^2^	27 (4)	28 (5)	31 (4)	26 (4)	**<0.0001**
FEV1%_pred_	107 (18)	77 (21)	102 (19)	68 (27)	**<0.0001**
FVC, %_pred_	103 (15)	88 (15)	95 (19)	92 (21)	**0.002**
FEV_1_/FVC, %	77 (6)	69 (13)	80 (6)	53 (12)	**<0.0001**
RV, %_pred_	98 (22)	121 (33)	100 (21)	148 (47)	**<0.0001**
TLC, %_pred_	100 (13)	102 (15)	96 (13)	113 (18)	**<0.0001**
RV/TLC, %_pred_	96 (17)	118 (23)	104 (16)	129 (26)	**<0.0001**
DL_CO_, %_pred_	90 (16)	–	87 (17)	61 (23)	**<0.0001**
*R*_aw_, %_pred_	83 (38)	105 (51)	65 (24)	117 (49)	**<0.0001**
*R*_5_, cmH_2_O·s/L	3.59 (1.68)	4.25 (1.49)	3.32 (1.12)	3.64 (1.23)	0.7
*R*_5‐19_, cmH_2_O·s/L	0.54 (0.76)	0.82 (0.87)	0.36 (0.54)	0.96 (0.79)	**0.001**
*X*_5_, cmH_2_O·s/L	−1.41 (0.88)	−1.86 (1.26)	−1.42 (0.72)	−2.41 (1.57)	**0.001**
*f*_res_, Hz^1^	19.77 (7.40)	19.53 (6.86)	20.20 (5.78)	23.66 (7.67)	**0.001**
*A*_*X*_, cmH_2_O/L	12.94 (14.94)	14.38 (14.90)	9.79 (7.57)	23.30 (19.96)	**0.008**
VDP %	3 (2)	5 (6)	5 (4)	19 (12)	**<0.0001**

SD = standard deviation; Sig diff = significance of difference; BMI = body mass index; FEV_1_ = forced expiratory volume in one‐second; %_pred_ = percent predicted; FVC = forced vital capacity; RV = residual volume; TLC = total lung capacity; DL_CO_ = diffusing capacity of the lung for carbon monoxide; *R*_aw_ = airways resistance measured using plethysmography; *R*_5_ = respiratory system resistance at 5 Hz; *R*_5‐19_ = frequency dependence of resistance; *X*_5_ = respiratory system reactance at 5 Hz; *f*_res_ = resonant frequency; *A*_*X*_ = reactance area; VDP = ventilation defect percent.

Pre‐bronchodilator values shown for never‐smokers and post‐bronchodilator values shown for asthmatics, ex‐smokers and COPD subjects.

* Significance of difference calculated using one‐way ANOVA for parametric variables and Kruskal–Wallis H test for nonparametric variables; significant values are bolded.

1 *n* = 42 for never‐smokers, *n* = 48 for asthma, *n* = 28 for ex‐smokers, *n* = 47 for COPD; *f*_res_ > 37 Hz for remaining subjects.

**Figure 2:**
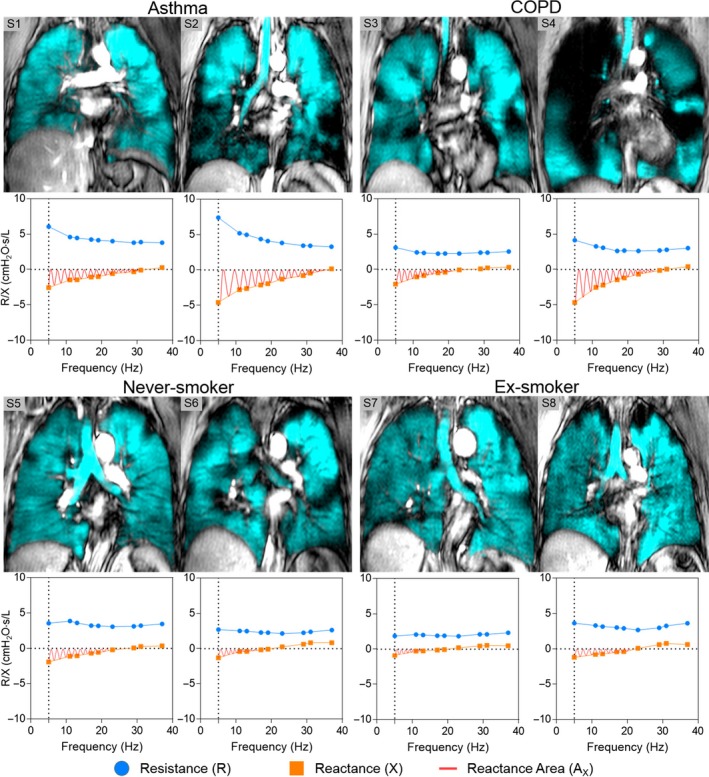
Relationship between MRI ventilation heterogeneity and impedance measurements in representative subjects. Centre slice coronal static ventilation ^3^He MRI (cyan) co‐registered to anatomical ^1^H (grey‐scale) and corresponding oscillometry plots for two representative asthma, COPD, never‐smoker and ex‐smoker participants. Worse ventilation heterogeneity qualitatively reflected increased frequency dependence of resistance and reactance as well as greater *A*_*X*_ in participants with asthma and COPD, but not for never‐smokers and ex‐smokers without COPD.

**Figure 3:**
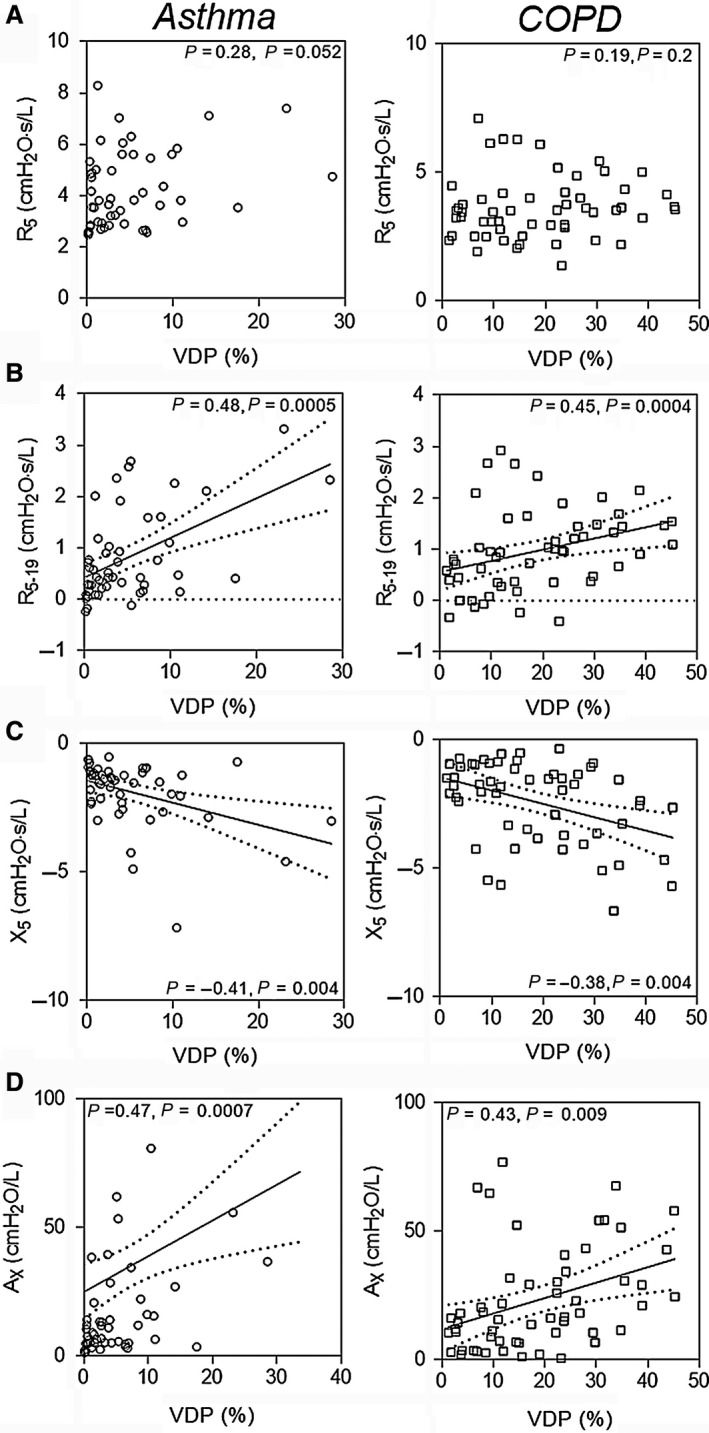
Quantitative relationships between MRI VDP and impedance measurements. (A) VDP was not significantly related to *R*_5_ in asthma nor in COPD subjects. (B) VDP was significantly related to *R*_5‐19_, and, (C) *X*_5_ and, (D) A_X_ in asthma and COPD participants.

Figure 2 shows ^3^He MRI ventilation defects and oscillometry plots for two representative participants in each group: one with low (normal) VDP and one with greater (abnormal) VDP. For participants with asthma and COPD, worse ventilation heterogeneity qualitatively reflected increased frequency dependence of resistance and reactance as well as greater *A*_*X*_. Increased ventilation heterogeneity in never‐smokers and ex‐smokers without COPD, however, did not reflect qualitatively apparent changes in oscillometry. As shown quantitatively in Figure 3, in asthma and COPD participants, post‐bronchodilator VDP was significantly related to *R*_5‐19_, *X*_5_, and *A*_*X*_, but not *R*_5_. For never‐smokers, VDP was significantly negatively related to *R*_5_ only and there were no relationships in ex‐smokers (not shown).

Table 2 shows multivariable models that predict VDP from oscillometric parameters *R*_5_, *R*_5‐19_, *X*_5_ and *A*_*X*_. *R*_5_ (*β *= −0.22, *P* = 0.01) and *X*_5_ (*β *= −0.34, *P* = 0.03) significantly added to the prediction of VDP for all subjects (Model 1: *R* = 0.63, *R* ^2^ = 0.39, *P* < 0.0001). For never‐smokers and ex‐smokers with and without COPD, *R*_5_ (*β *= −0.48, *P* = 0.001), *R*_5‐19_ (*β *= 0.35, *P* = 0.03), and *X*_5_ (*β *= −0.41, *P* = 0.03) significantly added to the prediction of VDP (Model 2: *R* = 0.66, *R* ^2^ = 0.44, *P* < 0.0001), whereas for only ex‐smokers with and without COPD, the overall model was significant (Model 3: *R* = 0.62, *R* ^2^ = 0.38, *P* < 0.0001) but none of the oscillometry parameters significantly added to the model. The overall model was also significant for asthmatic participants only (Model 4: *R* = 0.65, *R* ^2^ = 0.43, *P *= 0.001) but none of the oscillometry parameters significantly added to the model.

**Table 2:** Multivariable models to predict VDP from oscillometry

	Unstandardized		Standardized	*P*
Variable	B	Standard error	*β*	
MODEL 1: All subjects, *n* = 175 (*R* = 0.63; *R* ^2^ = 0.39, *P* < 0.0001)				
*R*_5_	−1.98	0.77	−0.22	**0.01**
*R*_5‐19_	1.81	1.61	0.15	0.3
*X*_5_	−2.75	1.26	−0.34	**0.03**
*A*_*X*_	0.13	0.11	0.21	0.2
MODEL 2: Never‐smokers, ex‐smokers with and without COPD, *n* = 126 (*R* = 0.66, *R* ^2^ = 0.44, *P* < 0.0001)				
*R*_5_	−3.96	1.17	−0.48	**0.001**
*R*_5‐19_	5.14	2.35	0.35	**0.03**
*X*_5_	−3.55	1.63	−0.41	**0.03**
*A*_*X*_	0.10	0.13	0.15	0.5
MODEL 3: Ex‐smokers with and without COPD, *n* = 84 (*R* = 0.62; *R* ^2^ = 0.38, *P* < 0.0001)				
*R*_5_	−2.95	1.93	−0.29	0.1
*R*_5‐19_	6.24	3.41	0.39	0.07
*X*_5_	−1.16	2.20	−0.14	0.6
*A*_*X*_	0.18	0.17	0.27	0.3
MODEL 4: Asthma only, *n* = 49 (*R* = 0.65, *R* ^2^ = 0.43, *P* = 0.001)				
*R*_5_	−1.28	0.84	−0.20	0.1
*R*_5‐19_	3.33	1.85	0.49	0.08
*X*_5_	1.15	1.36	0.24	0.4
*A*_*X*_	0.14	0.13	0.36	0.3

VDP = ventilation defect percent; *R*_5_ = resistance at 5 Hz; *R*_5‐19_ = resistance at 5 Hz minus resistance at 19 Hz; *X*_5_ = reactance at 5 Hz; *A*_*X*_ = reactance area.

Covariates: age, sex, BMI.

Of the 56 COPD participants evaluated, 33 had CT evidence of emphysema (RA_950_ ≥ 6.8%) (Gevenois et al. 1996) and 23 had no CT evidence of emphysema (RA_950_ < 6.8%). VDP was not significantly related to *R*_5_ regardless of the presence of emphysema, but VDP was related to *A*_*X*_ in COPD with (*ρ* = 0.39, *P* = 0.02) and without emphysema (*ρ *= 0.43, *P* = 0.04). VDP and *R*_5‐19_ were significantly related in COPD subjects without emphysema only (*ρ *= 0.54, *P* = 0.008), and significantly related to *X*_5_ in COPD subjects with emphysema only (*ρ *= −0.36, *P* = 0.04). There was no CT evidence of emphysema (all RA_950_ < 6.8%) in ex‐smokers without spirometry evidence of airflow limitation based on GOLD criteria of FEV_1_/FVC < 0.7 (Global Initiative for Chronic Obstructive Lung Disease (GOLD), 2017).

## Discussion

We evaluated oscillometry and hyperpolarized ^3^He MRI measurements in a relatively large group of patients with asthma and COPD as well as two control groups and made four important observations: (1) in asthma and COPD participants, VDP was significantly but weakly correlated with *R*_5‐19_, *X*_5_, and *A*_*X*_, but not *R*_5_, (2) in COPD patients without emphysema, VDP was related only to *R*_5‐19_ and *A*_*X*_, and only *X*_5_ and *A*_*X*_ in COPD patients with emphysema, (3) in an ex‐smoker control group, there were no significant relationships while in never‐smokers, only VDP and R_5_ were related, and, (4) *A*_*X*_ was weakly related to VDP in all subgroups with airflow obstruction, demonstrating its sensitivity to airflow obstruction but not specificity to type of obstruction.

The relationship between oscillometry and MRI VDP with quality‐of‐life measurements was previously investigated in 100 patients (Young et al. 2018) and this previous work was in agreement with our observations. The fact that there were no significant relationships between VDP and oscillometry in the control subgroups except for *R*_5_ and VDP in never‐smokers is also congruent with previous results (Young et al. 2018). Based on this previous work, our results were not unexpected. *R*_5_ reflects the resistance of the entire respiratory system including all airways (and not the just the small airways or the larger airways) and this may explain why significant relationships with VDP were not present. *R*_5_ was also not significantly different between the four subgroups, whereas *R*_5‐19_, *X*_5_, *A*_*X*_ as well as plethysmography‐measured airways resistance (*R*_aw_) were. This suggests that *R*_5_ is not sensitive to the differences in resistance in our patient population and this could be because much of the resistance in these patients may be due to the peripheral airways and this effect is overshadowed in the *R*_5_ signal. Oscillometry measurements that reflect the heterogeneity of airway narrowing (*R*_5‐19_) as well as *X*_5_ and *A*_*X*_ (Tgavalekos et al. 2007; Dellaca et al. 2009) were all related to VDP in asthma and all COPD patients, and none of these relationships were detected in never‐ or ex‐smokers. Notably, ventilation defects in severe COPD were previously shown to be related to both emphysema and small airways disease (Kirby et al. 2013; Capaldi et al. 2016) so the negative relationship between VDP and *X*_5_ in COPD was not surprising. This was not previously observed (Young et al. 2018) perhaps due to the current study's larger sample size across all grades of COPD severity. It has been shown in experimental studies in humans and animals however, that the major influence of heterogeneity is its impact on resistance and elastance between 0.1 Hz and 5 Hz (Hantos et al. 1986, 1990; Tepper et al. 1992), whereas our system is limited to 5 Hz and above. We are thus only capturing the “tail‐end” of the impact of heterogeneities using *R*_5‐19_ and this may explain the weak correlations observed.

To better understand how oscillometry and MRI VDP measurements are related and may explain the biomechanical impact of obstructive lung disease in patients, we generated multivariable models. We were surprised to observe that *R*_5_ significantly contributed to the models with all subjects (Model 1) and in never‐smokers and ex‐smokers with and without COPD (Model 2). *R*_5_ did not significantly contribute to the models in ex‐smokers with and without COPD (Model 3) or in asthmatics (Model 4). Based on these differences it is possible that the *R*_5_ results were being driven by the never‐smoker subgroup in whom there is no airflow obstruction. There were no significant coefficients in Model 3 and 4 which may be due to the smaller subgroup sizes which limited power to detect significant contributions. However, *R*_5‐19_ has the greatest relative influence on VDP in Models 3 and 4 which did not include the never‐smoker group.

COPD patients can be phenotyped based on the presence of airways disease and emphysema (Hogg et al. 2004) and these phenotypes also reflect differences in lung biomechanics and function (Wada et al. 2018). We observed differences in the relationships between VDP and oscillometry measurements in COPD patients with and without emphysema, although it is likely that all COPD patients had airways disease too. The fact that *X*_5_ and *A*_*X*_ were related to VDP in emphysematous COPD patients suggests that *X*_5_ and *A*_*X*_ may reflect parenchymal stiffness or derecruitment, resulting in ventilation defects. In contrast, in COPD patients with little or no emphysema, VDP was related to *A*_*X*_ and *R*_5‐19_ indicative of heterogeneous airway narrowing largely in the periphery, which was in agreement with previous work (Smith et al. 2005). The different behaviours of *R*_5‐19_ and *X*_5_ in COPD patients with and without emphysema suggests that *X*_5_ measures a different component that is independent of heterogeneous airway obstruction associated with *R*_5‐19_ (Otis et al. 1956). However, *A*_*X*_ was weakly significantly related to VDP in patients with and without emphysema, and this suggests that it is nonspecific to the type of obstruction (either airways disease or emphysema) in COPD patients. Emphysematous and airways disease phenotypes may be best identified by appropriate use of *R*_5‐19_ and *X*_5_. In COPD patients, it is also important to acknowledge that airways disease and emphysema phenotypes are typically observed in combination (Nakano et al. 2000), so future examinations should also evaluate mixed phenotypes which were not evaluated here.

*A_X_* was originally developed to improve the signal‐to‐noise ratio of respiratory system reactance compared to reactance values at a single frequency (Goldman 2001). Table 3 provides an overview of the advantages and limitations of oscillometry measurements of obstructive lung disease including *A_X_*. It is clear that *A_X_* is sensitive to airflow obstruction, however it is nonspecific to the type of obstruction and cannot distinguish airway constriction from lung recruitment or parenchymal stiffening. *R*_5‐19_ on the other hand is known to reflect obstruction in the distal airways (Grimby et al. 1968) whereas *X*_5_ is known to reflect elastic components of the lung. Moreover, *A*_*X*_ and the frequency dependence of resistance may depend on the number and choice of harmonics in the forcing waveform making them variable in different settings. For *A*_*X*_, the largest influence is the first harmonic since this is where the most of the area is located, and different commercially available devices start at different frequencies anywhere from 4 Hz for adults up to 8 Hz for children. Our data also demonstrated that for COPD participants with markedly abnormal *A_X_* greater than 50 cmH_2_O/L, VDP values ranged from 5% to 45% (Fig. 3D) and this suggests that *A*_*X*_ is weakly related to inter‐subject VDP differences. We note that *A*_*X*_ did not significantly contribute to VDP in any of the multivariable models. The multiple correlation coefficients ranged from 0.62 to 0.66 with *R* ^2^ = 0.39–0.44, so together, the oscillometry parameters contributed to no more than 44% of the variability in VDP regardless of subgroup.

**Table 3:** Advantages and limitations of oscillometry measurements

Advantages	Limitations
Frequency dependence of resistance (*R*_5‐19_)	
Signal averaging minimizes noise and potential artefacts Differentiates proximal from distal obstruction Detects mild/early obstruction	Variable in different settings
Reactance at 5 Hz (*X*_5_)	
Reflects elastic components Reflects peripheral airway disease	More noise Nonspecific to obstruction versus restriction
Reactance area (*A*_*X*_)	
Sensitive to obstruction Signal averaging minimizes noise and potential artefacts Units of cmH_2_O/L, similar to elastance Sensitive to intra‐subject response to therapy or provocation	Nonspecific to type of obstruction Variable in different settings When *f*_res_ is undefined, *A*_*x*_ value is user‐defined (hence variable between different devices) Weakly related to inter‐subject differences

We also recognize a number of other study limitations. Hyperpolarized ^3^He MRI is unlikely to be clinically used because of the vanishing global quantities and exorbitant cost of ^3^He (Shea and Morgan 2010). ^129^Xe MRI is more sensitive to airway obstruction (Kirby et al. 2013; Svenningsen et al. 2013), less costly and therefore, more feasible for clinical examinations so it will be important to compare oscillometry and ^129^Xe MRI measurements in patients. Moreover, shunting of the oscillatory waves to the upper airways reduces sensitivity to obstruction despite firm cheek‐holding (Cauberghs and van de Woestijne 1989). This means that in patients with obstruction, impedance may be underestimated, which may have also limited the correlation strengths observed here. We note that the never‐smoker control group studied here underwent testing without inhaled bronchodilators whereas asthmatics, ex‐smokers and COPD ex‐smokers were evaluated post‐bronchodilator. We previously showed that there was no post‐bronchodilator MRI ventilation response in elderly never‐smokers (Sheikh et al. 2014) with ventilation abnormalities, so we expect no confounding effects due to the lack of post‐bronchodilator measurements in this subgroup. Finally, we also acknowledge positional differences in the oscillometry (seated upright) and MRI measurements (supine). Respiratory system resistance is increased in the supine position compared to upright (Lorino et al. 1992; Gonzales et al. 2017) and the presence of emphysema also causes large upright to supine *A*_*X*_ variability (Dandurand et al. 2015), which may also explain why the relationships observed here were weak to moderate.

To our knowledge, this is the largest controlled evaluation of oscillometry and functional MRI undertaken in patients and healthy volunteers. The pattern of significant relationships for VDP with *R*_5‐19_ and *X*_5_ was different between the different disease subgroups (i.e., COPD with and without emphysema, asthma). On the other hand, the relationship of *A*_*X*_ with VDP was similar across disease subgroups, suggesting that *A_X_* is a sensitive but not specific measurement of obstruction. The different relationships for MRI VDP with *R*_5‐19_ and *X*_5_ may reflect airway and parenchymal disease‐specific biomechanical abnormalities that lead to ventilation defects.

## Conflict of Interest

GN Maksym is cofounder and scientific advisor to Thorasys (Montreal Canada) and less than 5% shareholder. Thorasys was not involved in the design, implementation or analysis of this work. The other authors have no conflicts of interest, financial or otherwise to declare.
